# Supplementary material for: Image-guided optogenetic spatiotemporal tissue patterning using μPatternScope
Source: Nat Commun. 2024 Dec 2;15:10469. doi: 10.1038/s41467-024-54351-6 (PMC11612157; doi:10.1038/s41467-024-54351-6)
Supplement: Supplementary file 1 — Supplementary Information [file 41467_2024_54351_MOESM1_ESM.pdf]

Supplementary Material

Image-Guided Optogenetic Spatiotemporal Tissue Patterning using μPatternScope

Sant Kumar, Hannes M. Beyer, Mingzhe Chen, Matias D. Zurbriggen, Mustafa Khammash

S1 μPatternScope hardware

S1.1 Parts list

The μPS hardware design mostly contains off-the-shelf and readily available components, making it easy to assemble and replicate with little expertise. Here is the detailed parts list for the assembly:

1. DMD evaluation board modules
   - DLPLCR65EVM (Qty. 1)and DLPLCRC900EVM (Qty. 1) – ordered from Texas Instruments
   - USB type A (male) to USB type B (male) cable (Qty. 1) – ordered from any relevant store
   - Power supply adapter (Qty. 1) – SDI65-12-U-P6 (ordered from DigiKey) or similar
2. Optical engine (Qty. 1) – SPARK Telecentric DLP650NIR (ordered from Optecks)
3. Projection lens (Qty. 1) – Nikon AF NIKKOR 50mm 1:1.8D camera lens (ordered from any relevant store)
4. Optical and mount components (Table S1) – ordered from Thorlabs
5. Heat sink for DMD (Made with Aluminium or Copper alloys – CAD file available in the GitHub repository `https://github.com/santkumar/uPatternScope`). Parts required to attach it with the DMD board (DLPLCR65EVM):
   - Screw (Qty. 4) – M3, 25mm long (shank length)
   - Washer (Qty. 4) – ID 3.2mm, OD 9mm (OD can be larger than 9mm)
   - Spring (Qty. 4) – 864840.0915 (ordered from BRW ToolShop) or similar
   - Heat sink compound paste (Qty. 1) – DC 340 100G (ordered from Distrelec) or similar
6. Microscope port adapter – Custom designed TI2-LA-BF adapter for Nikon Ti2E microscope (made with metal, preferable Aluminium, which can be anodized black) – CAD file available in the GitHub repository `https://github.com/santkumar/uPatternScope`). This adapter is required to interface μPS hardware with the microscope. We custom-designed this adapter for interfacing with Nikon Ti2E microscope. But, one can modify this CAD file to adapt to other microscope types accordingly.
7. DMD controller board holder – can be 3D printed with any material – CAD file available in the GitHub repository `https://github.com/santkumar/uPatternScope`).
8. HEX Keys (Metric) full set (tool kit)

S1.2 Assembly Instructions

For detailed assembly instructions and guidelines, please refer to the GitHub repository `https://github.com/santkumar/uPatternScope`.

Part number	Item	Qty
M450LP2	450nm 2118.1mW (Min) Mounted LED 2000mA	1
AD5LLG	Ø5 mm LLG to SM1 Adapter	2
XT34SM1	SM1 Adapter Face Plate for 34 mm Rails Four M3 Screws Included	1
C1515/M	Ø1.5" Mounting Post Bracket M6 Taps	1
PSHA/M	Adjustable Height Collar for Ø1.5" Posts M6 x 1.0 Locking Screw	1
PB4/M	Studded Pedestal Base Adapter M6 x 1.0 Thread	1
PF175B	Clamping Fork for Ø1.5" Pedestal Post or Post Pedestal Base Adapter Universal	1
LCP11/M	60 mm Cage Plate Internal and External SM2 Threads M4 Tap (One SM2RR Retaining Ring Included)	1
SM2L30C	SM2 Slotted Lens Tube 3" Thread Depth 2 Retaining Rings Included	1
SM2T20	SM2 (2.035"-40) Coupler External Threads 2" Long	1
LCP01/M	60 mm Cage Plate SM2 Threads 0.5" Thick M4 Tap (Two SM2RR Retaining Rings Included)	4
SM2L20C	SM2 Slotted Lens Tube 2" Thread Depth 2 Retaining Rings Included	1
SM2D25D	SM2 Ring-Actuated Iris Diaphragm (Ø1 - Ø25 mm)	1
LCP01B	60 mm Cage Mounting Bracket	1
TR150/M	Ø12.7 mm Optical Post SS M4 Setscrew M6 Tap L = 150 mm	1
UPH150/M	Ø12.7 mm Universal Post Holder Spring-Loaded Locking Thumbscrew L = 150 mm	1
LCPA1	60 mm Cage Alignment Plate with Ø1 mm Hole	1
ER12	Cage Assembly Rod 12" Long Ø6 mm	4
MB2025/M	Aluminum Breadboard 200 mm x 250 mm x 12.7 mm M6 Taps	1
P150/M	Ø1.5" Mounting Post M6 Taps L = 150 mm	1
P30/M	Ø1.5" Mounting Post M6 Taps L = 30 mm	1
LB1309-A	N-BK7 Bi-Convex Lens Ø2" f = 75.0 mm ARC: 350-700 nm	1
LB1630-A	N-BK7 Bi-Convex Lens Ø2" f = 100.0 mm ARC: 350-700 nm	1
LEDD1B	T-Cube LED Driver 1200 mA Max Drive Current (Power Supply Not Included)	1
SM1L10	SM1 Lens Tube 1.00" Thread Depth One Retaining Ring Included	1
KPS201	15 V 2.66 A Power Supply Unit with 3.5 mm Jack Connector for One K- or T-Cube	1
LLG05-4H	Liquid Light Guide Ø5 mm Core 340 - 800 nm 4' (1.2 m) Length	1

Table S1: μPatternScope assembly (optical and mount) components from **Thorlabs**

S2 μPatternScope software

All codes and instructions related to the μPS software suite are available in the GitHub repository `https://github.com/santkumar/uPatternScope`.

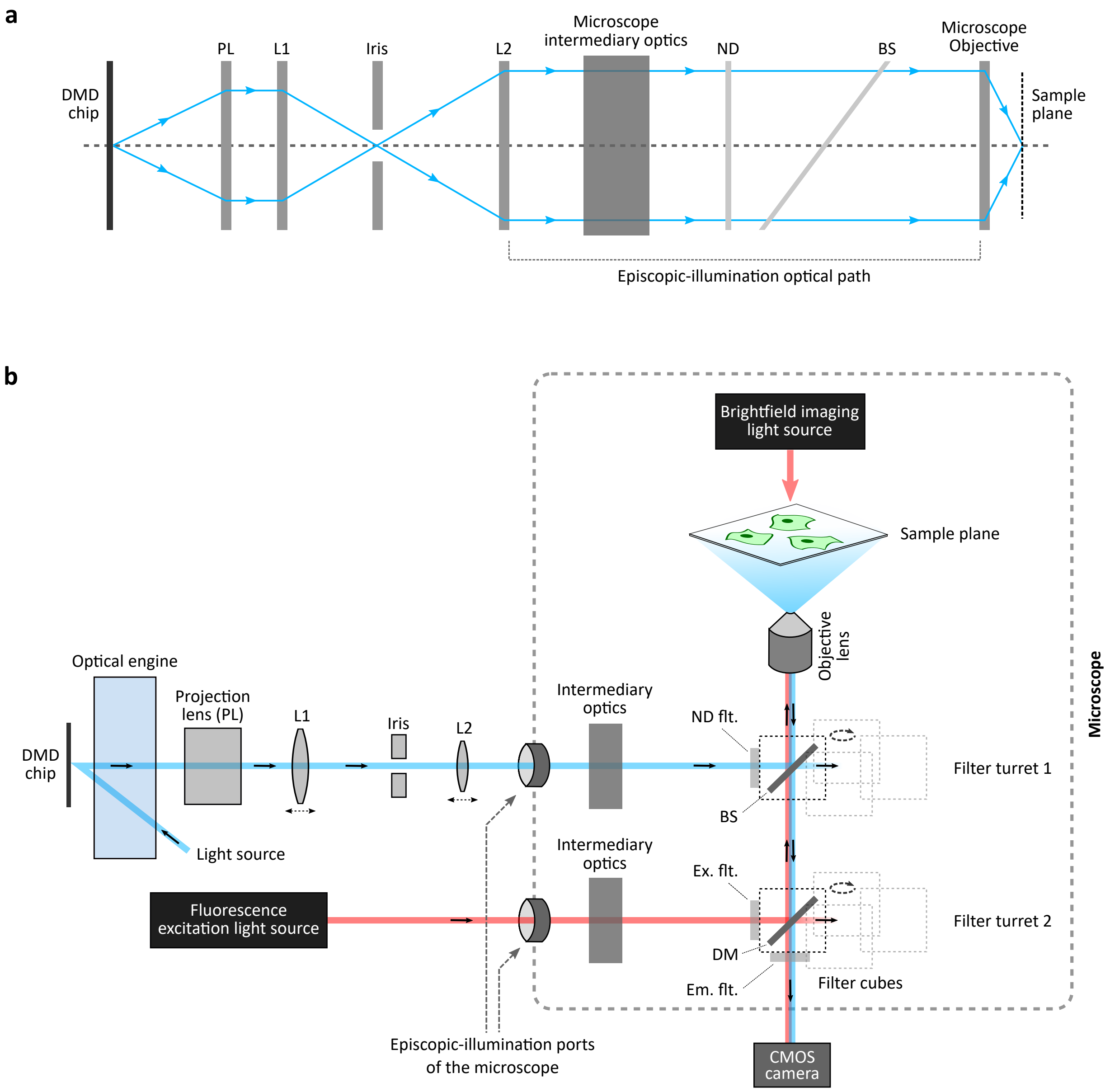

Supplementary Fig. S1. μPatternScope hardware optical configuration
Inspired by the microscopy-projection principles introduced in [1, 2], we devised a minimal optical configuration for the μPS hardware. **(a)** A representative ray diagram for the projection light path (PL = Nikon camera lens working as projection lens, L1 and L2 = biconvex lenses, ND = Natural density filter, BS = Beam splitter). The DMD chip and microscope sample plane are conjugate planes, that is, the image of DMD chip is focused on the sample plane. The iris is placed at an intermediate image plane, which allows varying the projection field of view and also assists with the initial alignment and tuning of the μPS optical path. ND filter (optional) and beam splitter are located in the episcopic-illumination optical path of the microscope. In some microscopes such as Nikon Ti2-E (used in our experiments), the episcopic path contains some intermediary optical elements (e.g. mirrors and lenses) present between L2 and the microscope objective. The microscope manufacturers have proprietary rights to these intermediate elements, and thus, their specifications are not disclosed. In such setups, light beams, after L2, need to be focused at a point on the optical axis before the objective. In our design, we have ensured sufficient space for the L2 mount, which one can slide to get the desired beam convergence after L2. **(b)** Beam path and optical setup used in our experiments (PL = Nikon camera lens working as projection lens, L1 and L2 = biconvex lenses, ND flt. = Natural density filter, BS = Beam splitter, Ex. flt. = Fluorescence imaging excitation filter, DM = Dichroic mirror, Em. flt. = Fluorescence imaging emission filter). In our experiments, we used a Nikon Ti2-E inverted widefield microscope with two parallel (double-layer configuration) episcopic illumination pathways. The bottom path solely serves fluorescence imaging excitation, while the top path guides μPS projection. During fluorescence imaging, filter turret 1 was rotated to an empty cassette, and filter turret 2 was rotated to the relevant imaging filter cube combination. For brightfield imaging, filter turret 1 was again rotated to an empty cassette, and filter turret 2 was rotated to a GFP filter set. For pattern projection via μPS, filter turret 1 was rotated to a filter cube with BS (50R/50T) and ND filters (optional) in the beam path. Additionally, the reflected projection light from the sample plane, after passing through the BS, was made to transmit through the filter turret 2, and then was finally captured by the microscope CMOS camera, which created the captured projection pattern image.

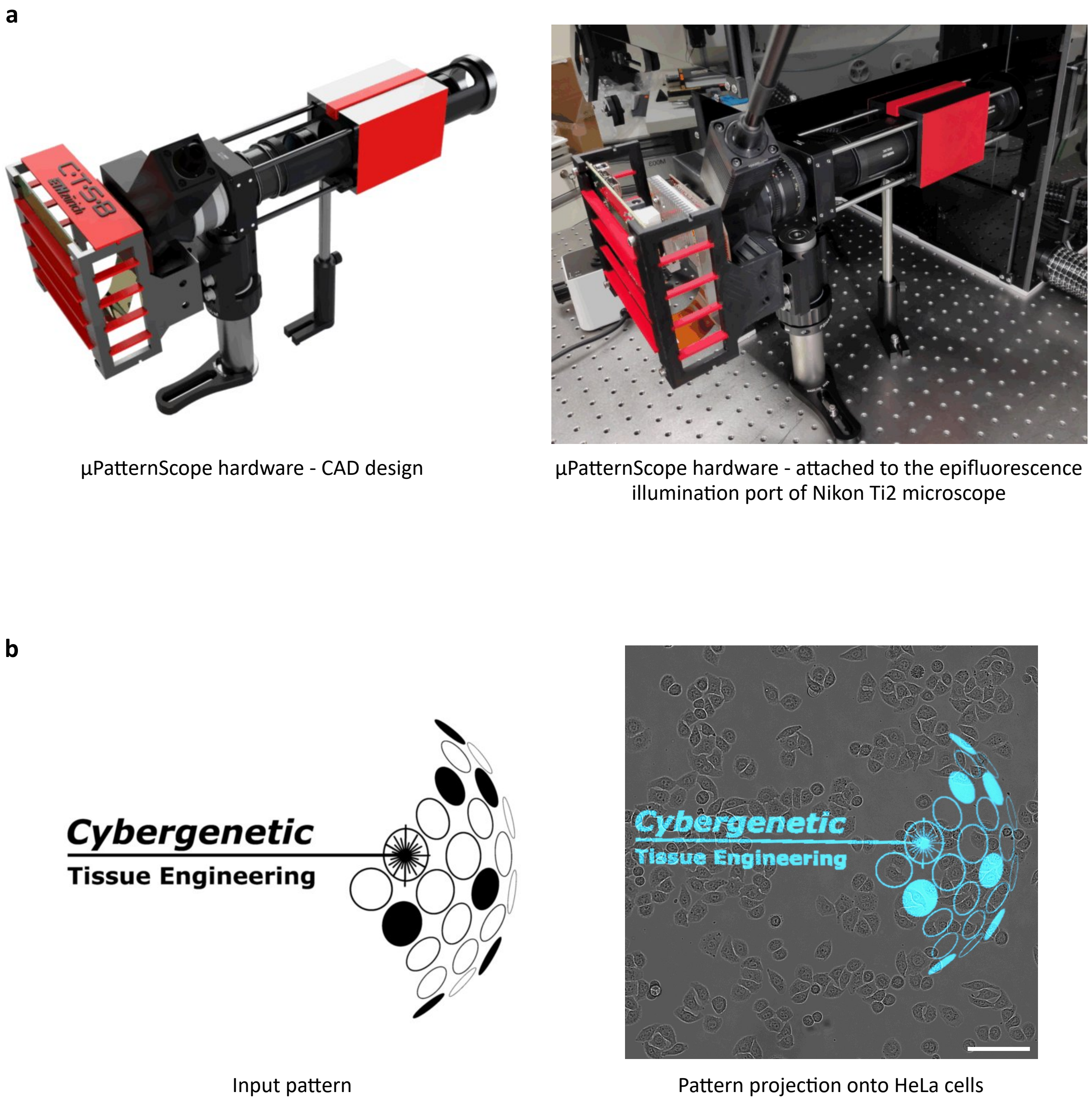

Supplementary Fig. S2. μPatternScope hardware and example projection
(a) CAD model of μPS hardware (left). μPS attached with the Nikon Ti2 microscope used in this work (right). **(b)** A given input image (left) is projected onto proliferating HeLa cells under the microscope (right) with 20X objective. Scale bar, 100μm.

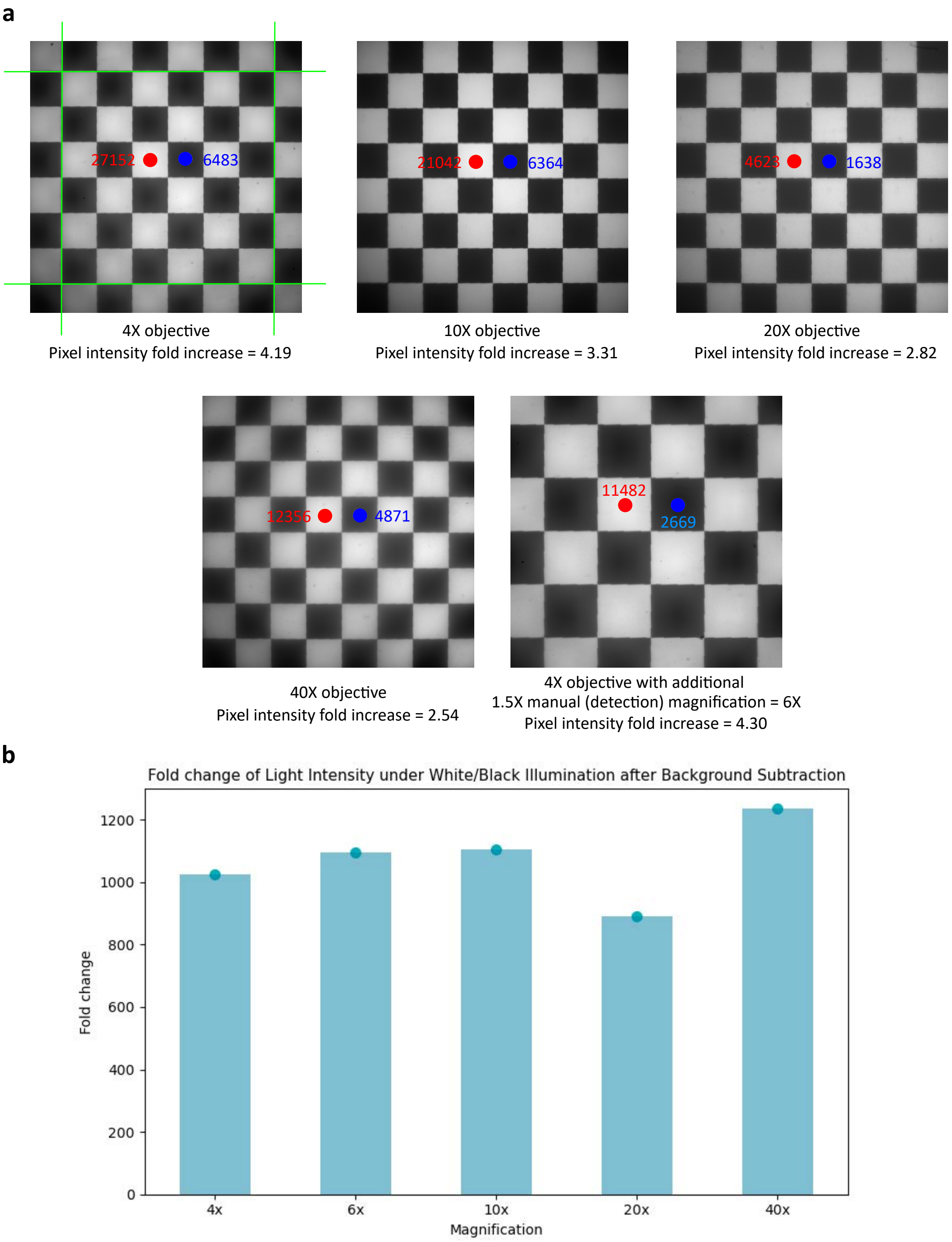

Supplementary Fig. S3. Checkerboard pattern projection and illumination fold change achieved via μPatternScope **(a)** A checkerboard pattern is projected onto the sample plane of Nikon Ti2 microscope via the attached μPS framework. The projection field of view under different objectives is captured via the microscope camera. The shown images are obtained after background subtraction. Green straight lines in the top left image show negligible distortion (even on the edges and corners of the microscope field of view) in the pattern projection via μPS. Pixel intensity fold increase is the fold change in the mean pixel intensity from blue circle (inside black square projection) to red circle (inside white square projection). Inset values are the respective mean pixel intensities for blue and red circles. **(b)** Two separate projection images are captured under the microscope, one with black image projection and another with white image projection on the whole field of view of the microscope. Here, fold change is calculated from mean pixel intensity in the black projection image to the white projection image captured via the microscope camera. Source data are provided as a Source Data file.

Note: Pixel intensities (and thus, fold change values) observed in the captured projection images (via microscope camera) depend not only on the input light illumination intensity on the DMD but, they also depend on camera parameters (e.g. exposure time, binning, etc.), any additional magnification (e.g. 1.5X shown in the bottom right panel of (a)) in the camera capture (detection) pathway of the microscope, illumination pattern (e.g. the size of squares in a checkerboard pattern), and microscope slide (where the patterns are being projected) properties (e.g. thickness, reflectivity, etc.).

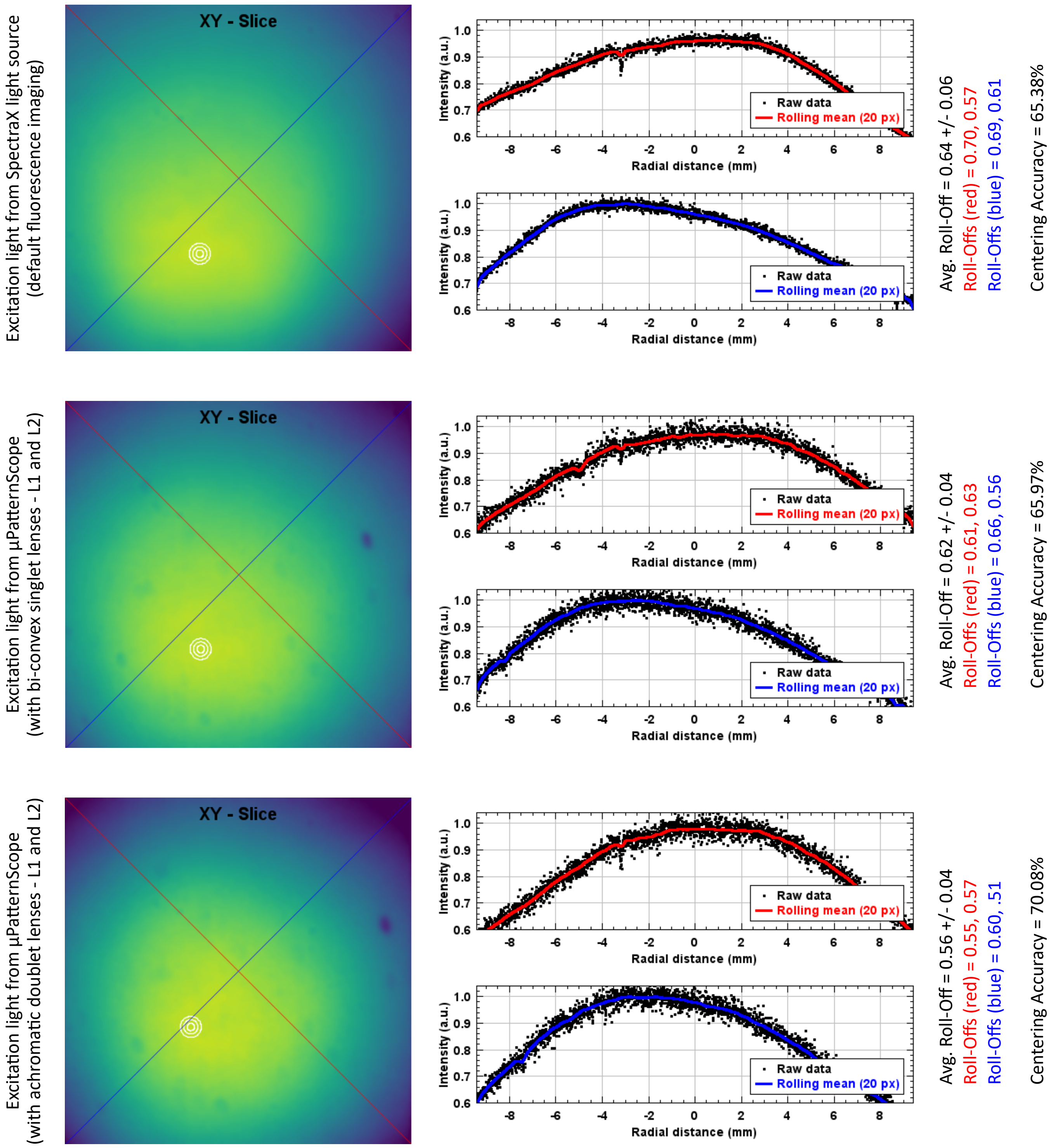

Supplementary Fig. S4. Uniformity test and analysis for the projection via μPatternScope
A uniform constant intensity pattern is projected via the μPS onto a fluorescent test slide placed on the microscope sample plane. The emitted fluorescence is captured via a YFP filter-set (543/22 nm emission filter, HC-BS520 beam splitter) and imaged with the microscope camera. Left side images represent the heat map of the camera-captured fluorescence in three settings: Top–fluorescence excitation via the default SpectraX light source in the microscope; Center–fluorescence excitation via the μPS with bi-convex singlet lenses (L1 and L2); Bottom–fluorescence excitation via the μPS with achromatic doublet lenses (L1 and L2). As observed in the fluorescence intensity profile (along diagonal lines) plots, the average roll-off with μPS excitation (0.62, 0.56) is similar to the default fluorescence excitation (0.64) in the microscope. This implies that the uniformity in sample illumination (in the microscope field of view) via μPS hardware is within the uniformity specification of the microscope. In this uniformity test/analysis, the microscope was configured with 4X objective and an additional 1.5X manual (detection) magnification (unless otherwise stated, this configuration was used in all experiments in this study). Roll-offs along a diagonal line in the fluorescence image are calculated by taking the ratio of fluorescence intensity at the corner to the maximum fluorescence intensity observed along the line.

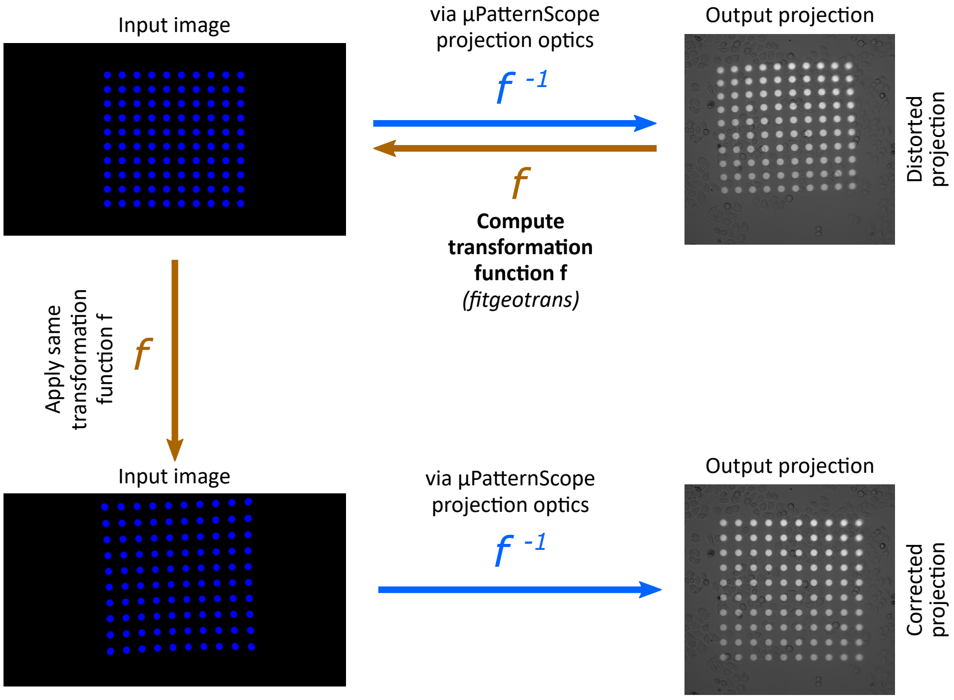

Supplementary Fig. S5. Software calibration for projection correction
An illustration of the software calibration performed for identifying the mapping (transformation function f) between camera pixels (in the captured projection image) and DMD pixels (in the input image) when the given input image is projected via μPS hardware onto the microscope sample plane. This mapping is then used to modify any input pattern to get the desired corrected projection pattern.

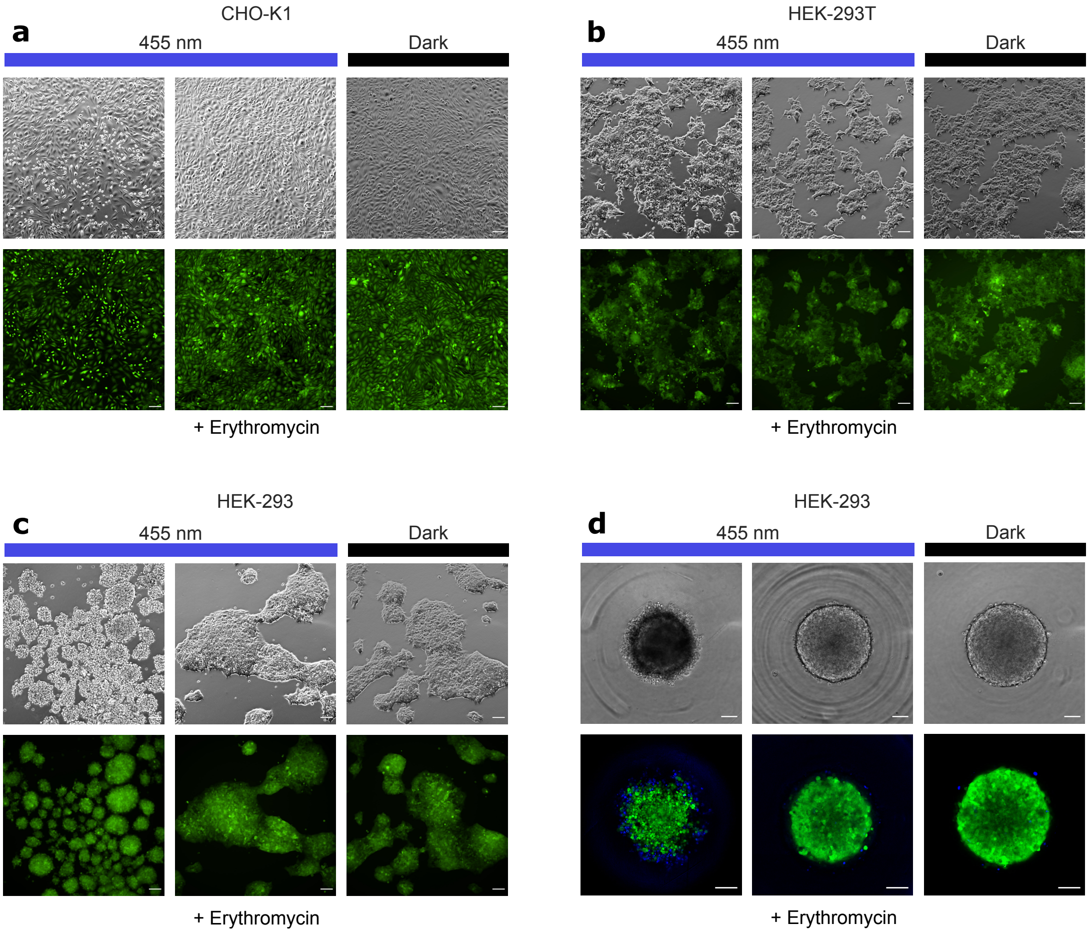

Supplementary Fig. S6. Test for blue light induction of apoptosis in mixed cultures of genomically-engineered cells. **(a)** CHO-K1, **(b)** HEK-293T, and **(c)** HEK-293 mixed cultures obtained from antibiotic selection upon genomic engineering with the vectors shown in Figure 2c. **(d)** Induction of *revCASP3* expression and apoptosis in 3D spheroid cultures generated from the cells in (c). SYTOX Blue death cell stain is shown in blue. **(a-d)** Cell cultures were cultivated for 24 h under 455 nm blue light for the induction of *revCASP3* expression and test for apoptosis. Alternatively, the cells were protected by the addition of 2 $\mu g\, mL^{-1}$ erythromycin or were kept in darkness. Scale bar, 100 μm. Experiments corresponding to (a)(b)(c) were performed once with three replicates per condition. Experiment corresponding to (d) was performed twice with three replicates per condition and similar results.

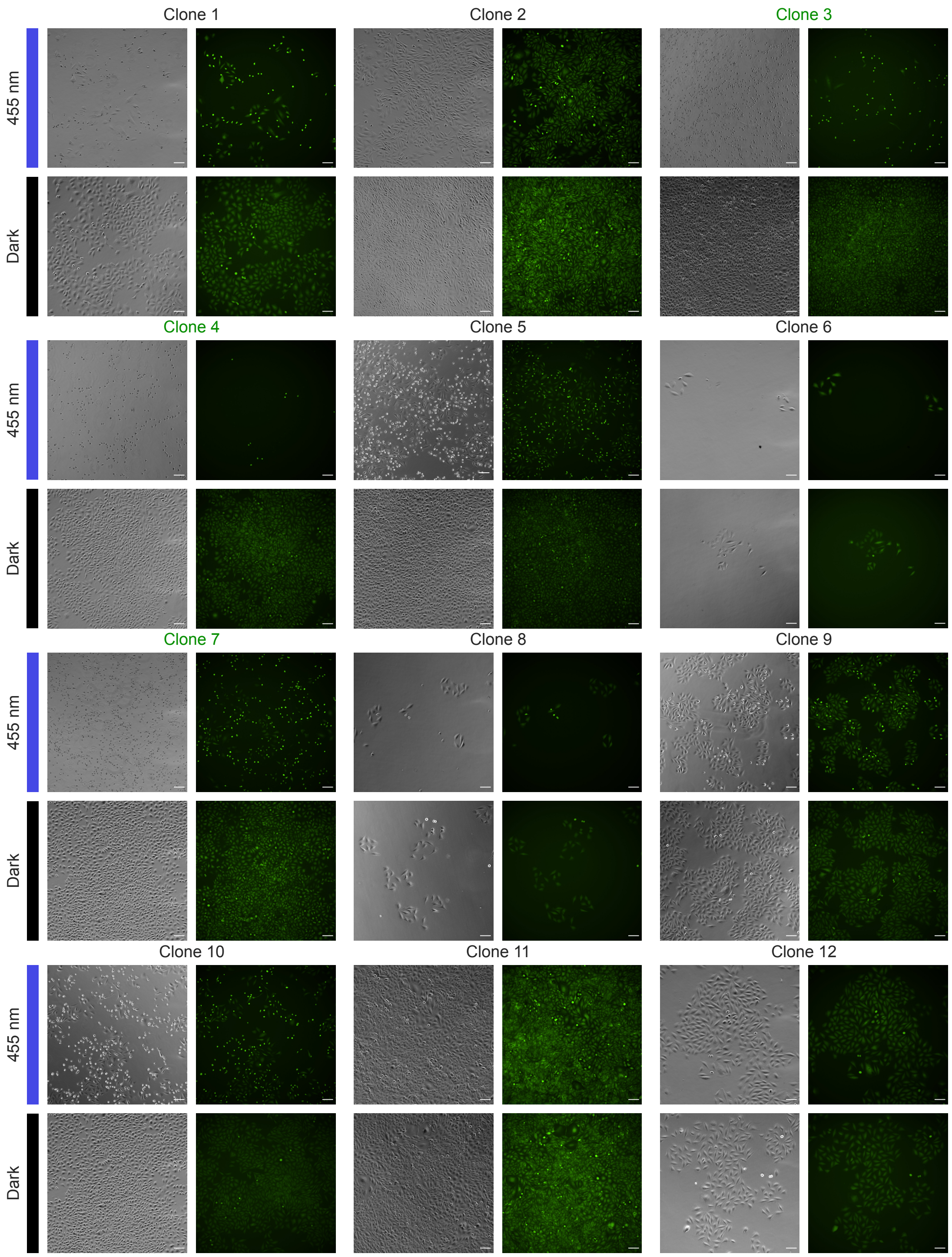

Clone 1
Clone 2
Clone 3
455 nm
Dark
Clone 4
Clone 5
Clone 6
455 nm
Dark
Clone 7
Clone 8
Clone 9
455 nm
Dark
Clone 10
Clone 11
Clone 12
455 nm
Dark

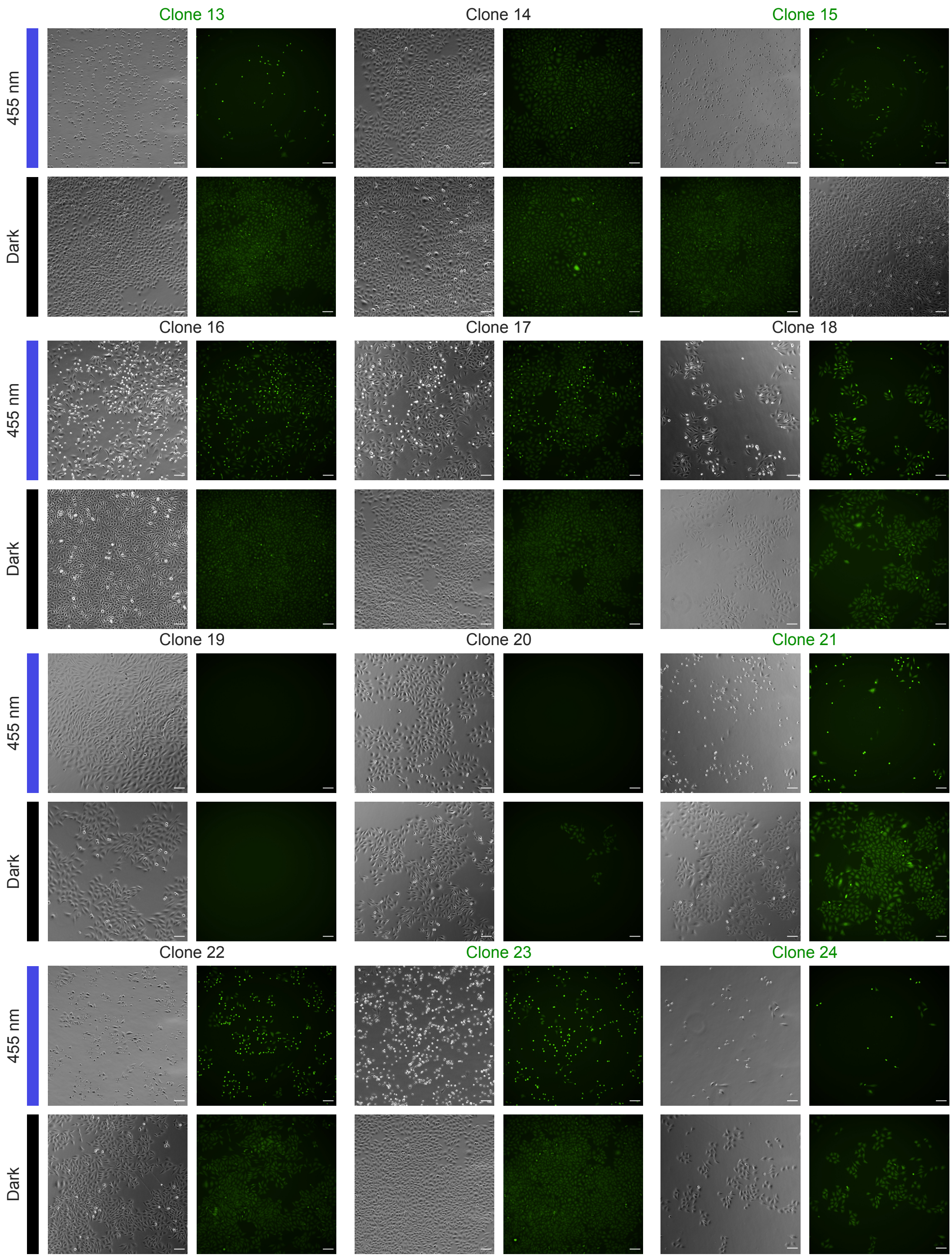
Clone 13
Clone 14
Clone 15
Clone 16
Clone 17
Clone 18
Clone 19
Clone 20
Clone 21
Clone 22
Clone 23
Clone 24
455 nm
Dark

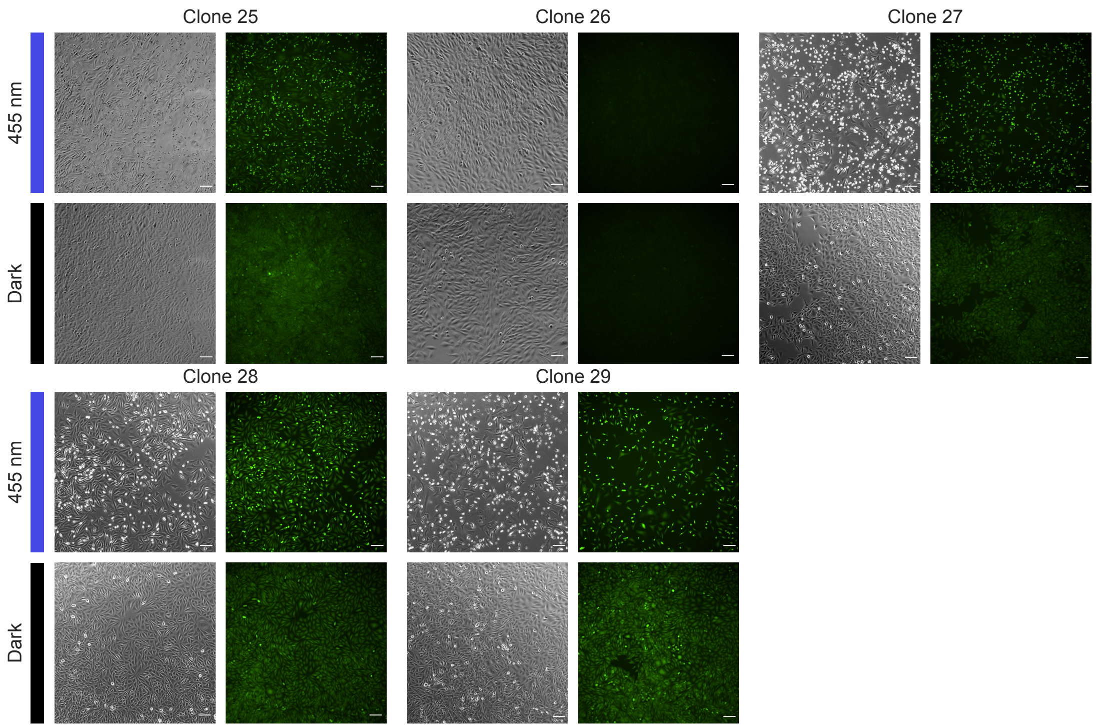

Supplementary Fig. S7. Clonal selection of CHO-K1$^{\mathbf{ApOpto}}$ cells. 29 clones were randomly selected and tested for the induction of cell death by illumination with 455 nm blue light for 24 h at an intensity of 10 $\mu mol\, m^{-2}\, s^{-1}$. Brightfield and GFP images are shown. Scale bar, 100 μm. The eight best-performing clones are indicated in green. Clone # 4 represents the CHO-K1^{ApOpto} cell line; this acquisition is also shown in Figure 2d. This experiment was performed once for screening purpose.

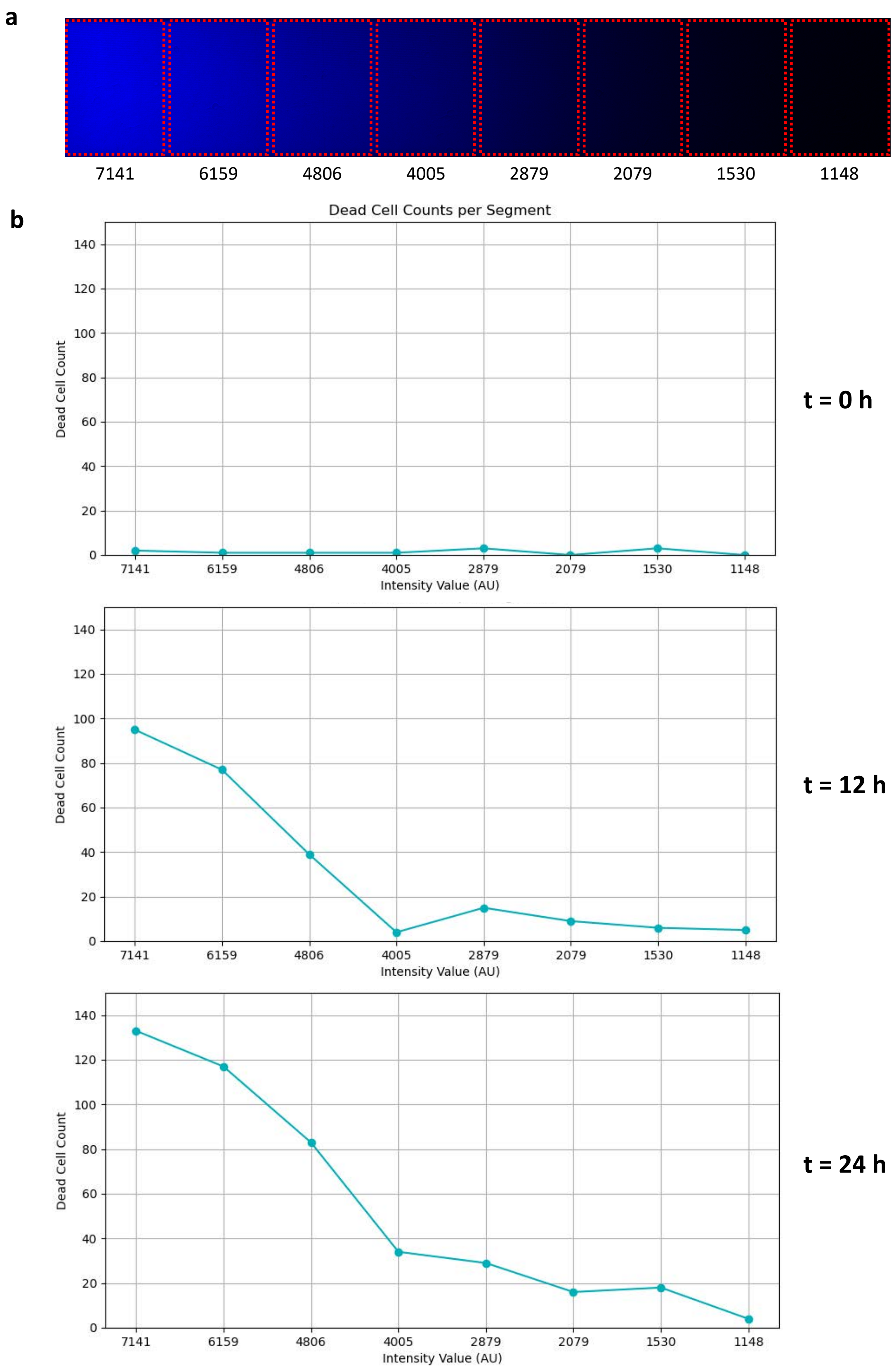

Supplementary Fig. S8. Continuous blue light intensity gradient illumination over CHO-K1$^{\mathbf{ApOpto}}$ cells via the μPS (Quantification of Figure 3d). (a) The gradient intensity projection is divided into 8 rectangular segments for quantification. Inset values are mean pixel intensities in the corresponding segments. **(b)** Number of dead cells within each segment is calculated based on the SYTOX blue (dead cell stain) image of the respective segment (by segmenting dead cells using fastER [3]). Significant apoptosis is observed at higher blue-light illumination intensities (at 4806 and higher intensities here) even at 12 hours of constant illumination. Source data are provided as a Source Data file.

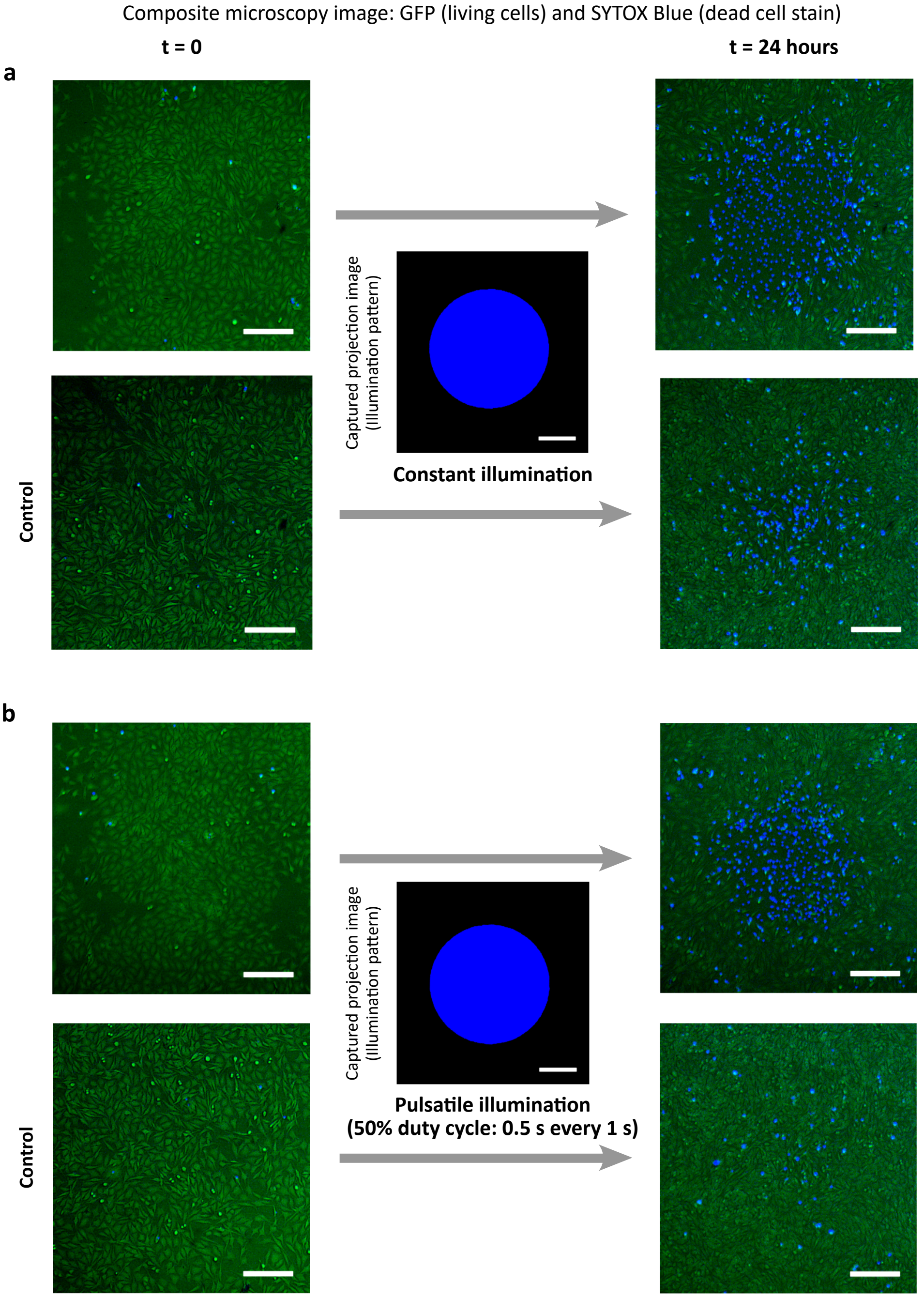

Supplementary Fig. S9. Optogenetic induction of apoptosis with pulsatile blue light illumination. **(a)** Constant pattern projection onto CHO-K1^{ApOpto} cells (top image panels) for 24 h using the μPS hardware. The bottom image panels show the control experiment using the parental CHO-K1 cell line lacking the engineered optogenetic apoptosis circuit (Figure 2). **(b)** The same illumination pattern as in a) is projected in pulses of 0.5 seconds ON intervals every second for a total duration of 24 h. Here, both continuous and pulsatile illumination regimes can induce apoptosis patterns in CHO-K1^{ApOpto} cell sheets. As observed in control experiments, the used blue light illumination intensity (blue light illumination irradiance, $\sim$1000 μW/cm^2) is phototoxic to the target cells under continuous illumination. The same light intensity can be used in the pulsatile illumination regime to reduce cell death due to phototoxicity. Scale bar, 200 μm. Experiments corresponding to (a) and (b) were performed once.

References

[1] Peixin Zhu, Otto Fajardo, Jennifer Shum, Yan-Ping Zhang Schärer, and Rainer W Friedrich. High-resolution optical control of spatiotemporal neuronal activity patterns in zebrafish using a digital micromirror device. *Nature protocols*, 7(7):1410–1425, 2012.

[2] Marc Rullan, Dirk Benzinger, Gregor W Schmidt, Andreas Milias-Argeitis, and Mustafa Khammash. An optogenetic platform for real-time, single-cell interrogation of stochastic transcriptional regulation. *Molecular cell*, 70 (4):745–756, 2018.

[3] Oliver Hilsenbeck, Michael Schwarzfischer, Dirk Loeffler, Sotiris Dimopoulos, Simon Hastreiter, Carsten Marr, Fabian J Theis, and Timm Schroeder. faster: a user-friendly tool for ultrafast and robust cell segmentation in large-scale microscopy. *Bioinformatics*, 33(13):2020–2028, 2017.
