# Supplementary material for: Image-guided optogenetic spatiotemporal tissue patterning using μPatternScope
Source: Nat Commun. 2024 Dec 2;15:10469. doi: 10.1038/s41467-024-54351-6 (PMC11612157; doi:10.1038/s41467-024-54351-6)
Supplement: Supplementary file 10 — Reporting Summary [file 41467_2024_54351_MOESM10_ESM.pdf]

Corresponding author(s): Mustafa Khammash, Matias D. Zurbriggen

Last updated by author(s): Oct 18, 2024

Reporting Summary

Nature Portfolio wishes to improve the reproducibility of the work that we publish. This form provides structure for consistency and transparency in reporting. For further information on Nature Portfolio policies, see our Editorial Policies and the Editorial Policy Checklist.

Statistics

For all statistical analyses, confirm that the following items are present in the figure legend, table legend, main text, or Methods section.

n/a	Confirmed	
☐	☒	The exact sample size (*n*) for each experimental group/condition, given as a discrete number and unit of measurement
☒	☐	A statement on whether measurements were taken from distinct samples or whether the same sample was measured repeatedly
☒	☐	The statistical test(s) used AND whether they are one- or two-sided<br>*Only common tests should be described solely by name; describe more complex techniques in the Methods section.*
☒	☐	A description of all covariates tested
☒	☐	A description of any assumptions or corrections, such as tests of normality and adjustment for multiple comparisons
☒	☐	A full description of the statistical parameters including central tendency (e.g. means) or other basic estimates (e.g. regression coefficient) AND variation (e.g. standard deviation) or associated estimates of uncertainty (e.g. confidence intervals)
☒	☐	For null hypothesis testing, the test statistic (e.g. *F*, *t*, *r*) with confidence intervals, effect sizes, degrees of freedom and *P* value noted<br>*Give P values as exact values whenever suitable.*
☒	☐	For Bayesian analysis, information on the choice of priors and Markov chain Monte Carlo settings
☒	☐	For hierarchical and complex designs, identification of the appropriate level for tests and full reporting of outcomes
☒	☐	Estimates of effect sizes (e.g. Cohen's *d*, Pearson's *r*), indicating how they were calculated

Our web collection on statistics for biologists contains articles on many of the points above.

Software and code

Policy information about availability of computer code

Data collection	1. YouScope R2020 (build: 2.2.0, open source) -- Used for microscope and projector control.<br>2. MATLAB R2020b (academic use) -- Used for µPatternScope software development.<br>3. Autodesk Fusion (education license) -- Used for computer-aided design of µPatternScope hardware assembly.<br><br>All µPatternScope CAD designs, detailed assembly instructions, software suite codes, and details are provided in the GitHub repository https://github.com/santkumar/uPatternScope.
Data analysis	All analyses in this study were performed on MATLAB R2020b (academic use) or Jupyter Notebook platform using custom scripts. Microscopy images were fused and formatted using MATLAB R2020b (academic use) and ImageJ 1.53t. Manuscript figures were structured and formatted on Inkscape (v0.92 and v1.2, open source).

For manuscripts utilizing custom algorithms or software that are central to the research but not yet described in published literature, software must be made available to editors and reviewers. We strongly encourage code deposition in a community repository (e.g. GitHub). See the Nature Portfolio guidelines for submitting code & software for further information.

Data

Policy information about availability of data

All manuscripts must include a data availability statement. This statement should provide the following information, where applicable:

- Accession codes, unique identifiers, or web links for publicly available datasets
- A description of any restrictions on data availability
- For clinical datasets or third party data, please ensure that the statement adheres to our policy

All relevant data and captured images are available in the manuscript figures. Source data are provided with this paper. DNA samples are available on request.

Research involving human participants, their data, or biological material

Policy information about studies with human participants or human data. See also policy information about sex, gender (identity/presentation), and sexual orientation and race, ethnicity and racism.

Reporting on sex and gender	*Use the terms sex (biological attribute) and gender (shaped by social and cultural circumstances) carefully in order to avoid confusing both terms. Indicate if findings apply to only one sex or gender; describe whether sex and gender were considered in study design; whether sex and/or gender was determined based on self-reporting or assigned and methods used. Provide in the source data disaggregated sex and gender data, where this information has been collected, and if consent has been obtained for sharing of individual-level data; provide overall numbers in this Reporting Summary. Please state if this information has not been collected. Report sex- and gender-based analyses where performed, justify reasons for lack of sex- and gender-based analysis.*
Reporting on race, ethnicity, or other socially relevant groupings	*Please specify the socially constructed or socially relevant categorization variable(s) used in your manuscript and explain why they were used. Please note that such variables should not be used as proxies for other socially constructed/relevant variables (for example, race or ethnicity should not be used as a proxy for socioeconomic status). Provide clear definitions of the relevant terms used, how they were provided (by the participants/respondents, the researchers, or third parties), and the method(s) used to classify people into the different categories (e.g. self-report, census or administrative data, social media data, etc.) Please provide details about how you controlled for confounding variables in your analyses.*
Population characteristics	*Describe the covariate-relevant population characteristics of the human research participants (e.g. age, genotypic information, past and current diagnosis and treatment categories). If you filled out the behavioural & social sciences study design questions and have nothing to add here, write "See above."*
Recruitment	*Describe how participants were recruited. Outline any potential self-selection bias or other biases that may be present and how these are likely to impact results.*
Ethics oversight	*Identify the organization(s) that approved the study protocol.*

Note that full information on the approval of the study protocol must also be provided in the manuscript.

Field-specific reporting

Please select the one below that is the best fit for your research. If you are not sure, read the appropriate sections before making your selection.

☒ Life sciences ☐ Behavioural & social sciences ☐ Ecological, evolutionary & environmental sciences

For a reference copy of the document with all sections, see nature.com/documents/nr-reporting-summary-flat.pdf

Life sciences study design

All studies must disclose on these points even when the disclosure is negative.

Sample size	In this work, we were targeting 2D layer of cells placed under the microscope with blue light patterns using our uPatternScope framework. The starting cell confluency for such an experiment was carefully chosen with respect to experiment duration, in order to avoid over-growth of cells hindering uniform exposure. Microscopy field of view (FOV) over the cells was chosen with enough number of uniformly spread target cells so that an observable pattern of apoptosis could be manifested in the FOV.
Data exclusions	No data were excluded from analyses in this work. However, a couple of experiments were rejected either due to a very low starting cell confluency (leading to insufficient number of target cells needed to generate observable patterns in the microscopy field of view) or due to a high starting cell confluency (leading to over-growth of cells during pattern projection duration).
Replication	The functionality of the uPatternScope framework was repeatedly validated throughout the different experiments and characterizations shown in the main text and in the Supplementary Information. The here-developed optogenetic cell line was repeatedly tested in the different experiments throughout the study. The design concept was verified in other cell lines described in the Supplementary Information.
Randomization	The cells for a given patterning experiment were chosen and seeded randomly into microscopy-compatible culture plates. In that culture

	plate, a specific well and a specific microscopy field of view (FOV) was chosen based on the spatial arrangement of cells (favoring FOVs where cells were uniformly spread with sufficient confluency).
Blinding	No blinding was performed.

Reporting for specific materials, systems and methods

We require information from authors about some types of materials, experimental systems and methods used in many studies. Here, indicate whether each material, system or method listed is relevant to your study. If you are not sure if a list item applies to your research, read the appropriate section before selecting a response.

Materials & experimental systems

n/a	Involved in the study
☒	☐ Antibodies
☐	☒ Eukaryotic cell lines
☒	☐ Palaeontology and archaeology
☒	☐ Animals and other organisms
☒	☐ Clinical data
☒	☐ Dual use research of concern
☒	☐ Plants

Methods

n/a	Involved in the study
☒	☐ ChIP-seq
☒	☐ Flow cytometry
☒	☐ MRI-based neuroimaging

Eukaryotic cell lines

Policy information about cell lines and Sex and Gender in Research

Cell line source(s)	Chinese hamster ovary CHO-K1 (DSMZ, ACC 110)<br>Human embryonic kidney HEK-293 (DSMZ, ACC 305)<br>Human embryonic kidney HEK-293T (DSMZ, ACC 635)<br>HeLa (ATCC, CCL 2)
Authentication	Cell line information available at:<br>American Type Culture Collection (ATCC), www.atcc.org<br>German Collection of Microorganisms and Cell Cultures (DSMZ), Braunschweig, Germany, www.dsmz.de<br>Cell lines were authenticated by ATCC and DSMZ, not by us.
Mycoplasma contamination	The used/generated cell lines were not routinely tested for for mycoplasma contamination.
Commonly misidentified lines<br>(See ICLAC register)	No commonly misidentified cell lines were used in this study.

Plants

Seed stocks	*Report on the source of all seed stocks or other plant material used. If applicable, state the seed stock centre and catalogue number. If plant specimens were collected from the field, describe the collection location, date and sampling procedures.*
Novel plant genotypes	*Describe the methods by which all novel plant genotypes were produced. This includes those generated by transgenic approaches, gene editing, chemical/radiation-based mutagenesis and hybridization. For transgenic lines, describe the transformation method, the number of independent lines analyzed and the generation upon which experiments were performed. For gene-edited lines, describe the editor used, the endogenous sequence targeted for editing, the targeting guide RNA sequence (if applicable) and how the editor was applied.*
Authentication	*Describe any authentication procedures for each seed stock used or novel genotype generated. Describe any experiments used to assess the effect of a mutation and, where applicable, how potential secondary effects (e.g. second site T-DNA insertions, mosiacism, off-target gene editing) were examined.*
