# Supplementary material for: Image-guided optogenetic spatiotemporal tissue patterning using μPatternScope
Source: Nat Commun. 2024 Dec 2;15:10469. doi: 10.1038/s41467-024-54351-6 (PMC11612157; doi:10.1038/s41467-024-54351-6)
Supplement: Supplementary file 11 — Description of Additional Supplementary Files [file 41467_2024_54351_MOESM11_ESM.pdf]

Description of Additional Supplementary Files

File Name: Supplementary Movie 1

Description: μPS hardware. CAD rendered video of the μPS hardware, showing the assembly of optical components and mounts. Please refer to Figure 1a for the μPS hardware design sketch.

File Name: Supplementary Movie 2-4

Description: Continuous blue light intensity gradient illumination over CHO-K1^{ApOpto} cells via the μPS. 24 hours time-lapse microscopy imaging video (Supplementary Movie 2: Brightfield imaging, Supplementary Movie 3: SYTOX Blue imaging, Supplementary Movie 4: GFP imaging) of the experiment. The illuminated blue light gradient pattern is shown in Figure 3d.

File Name: Supplementary Movie 5-8

Description: “Apoptotic” tic-tac-toe game using the μPS framework and CHO-K1^{ApOpto} cells. Time-lapse microscopy images (Supplementary Movie 7: Captured projection imaging, Supplementary Movie 8: SYTOX Blue imaging) of the raster-field of a 40-hour game experiment, where computer player - A (playing circle pattern) won. Time-lapse microscopy images (Supplementary Movie 5: Captured projection imaging, Supplementary Movie 6: SYTOX Blue imaging) of the raster-field of a 50-hour “tie” game experiment. Please refer to Figure 5 for detailed description.
